# An Annotated Genome for *Haliotis rufescens* (Red Abalone) and Resequenced Green, Pink, Pinto, Black, and White Abalone Species

**DOI:** 10.1093/gbe/evz006

**Published:** 2019-01-17

**Authors:** Rick E Masonbrink, Catherine M Purcell, Sara E Boles, Andrew Whitehead, John R Hyde, Arun S Seetharam, Andrew J Severin

**Affiliations:** 1 Genome Informatics Facility, Iowa State University; 2 Ocean Associates, Inc. Under Contract to NOAA Fisheries, Southwest Fisheries Science Center, La Jolla, California; 3 Department of Environmental Toxicology, University of California, Davis; 4 NOAA Fisheries, Southwest Fisheries Science Center, La Jolla, California

**Keywords:** red abalone, genome assembly, aquaculture, *Haliotis*, *Haliotis rufescens*

## Abstract

Abalone are one of the few marine taxa where aquaculture production dominates the global market as a result of increasing demand and declining natural stocks from overexploitation and disease. To better understand abalone biology, aid in conservation efforts for endangered abalone species, and gain insight into sustainable aquaculture, we created a draft genome of the red abalone (*Haliotis rufescens*). The approach to this genome draft included initial assembly using raw Illumina and PacBio sequencing data with MaSuRCA, before scaffolding using sequencing data generated from Chicago library preparations with HiRise2. This assembly approach resulted in 8,371 scaffolds and total length of 1.498 Gb; the N50 was 1.895 Mb, and the longest scaffold was 13.2 Mb. Gene models were predicted, using MAKER2, from RNA-Seq data and all related expressed sequence tags and proteins from NCBI; this resulted in 57,785 genes with an average length of 8,255 bp. In addition, single nucleotide polymorphisms were called on Illumina short-sequencing reads from five other eastern Pacific abalone species: the green (*H. fulgens*), pink (*H. corrugata*), pinto (*H. kamtschatkana*), black (*H. cracherodii*), and white (*H. sorenseni*) abalone. Phylogenetic relationships largely follow patterns detected by previous studies based on 1,784,991 high-quality single nucleotide polymorphisms. Among the six abalone species examined, the endangered white abalone appears to harbor the lowest levels of heterozygosity. This draft genome assembly and the sequencing data provide a foundation for genome-enabled aquaculture improvement for red abalone, and for genome-guided conservation efforts for the other five species and, in particular, for the endangered white and black abalone.

## Introduction

**Fig. 1.**
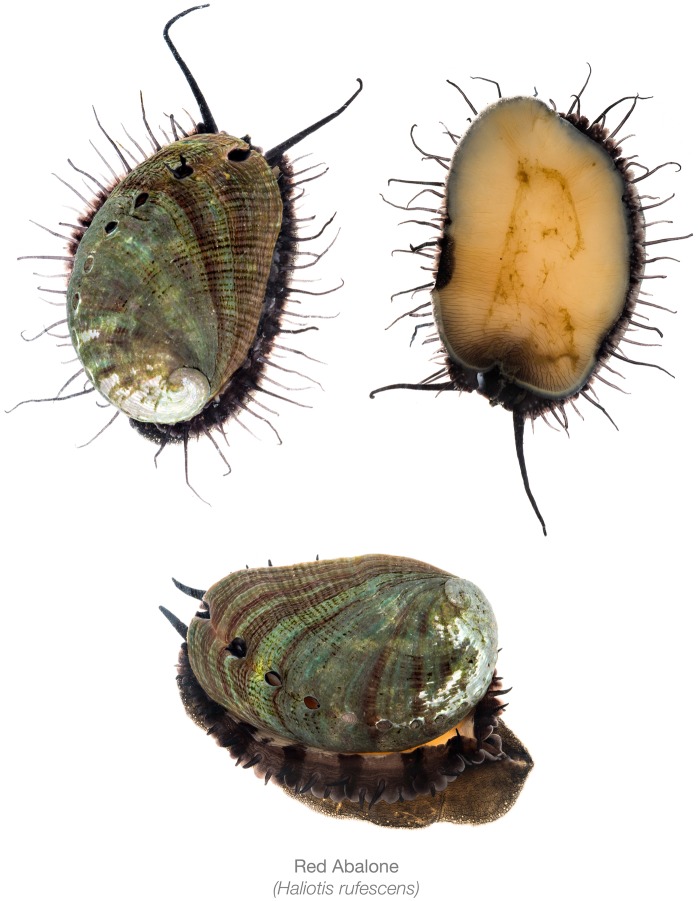
—Photograph of *Haliotis rufescens* by Michael Ready in collaboration with the NOAA Fisheries, Southwest Fisheries Science Center, Fisheries Resources Division, and the California Department of Fish and Wildlife.

Approximately 362 metric tons of farmed abalone are produced annually in the United States (Cook 2016). The dominantly cultured species on the west coast of California is red abalone (*Haliotis rufescens*, fig. 1), which grows quickly and reaches a large size in culture; popular in the US market, they are also one of the most valuable species in the mollusc industry (Aguilar-Espinoza et al. 2014; Brokordt et al. 2015). Of commercially cultured abalone on the west coast, *H. rufescens* is the most temperate with a range extending from Oregon to Baja California (Geiger 1999). Green (*H. fulgens*) and pink abalone (*H. corrugata*) have more southern distributions and are of greater interest for aquaculture production in Mexico, where they can be grown at warmer water temperatures (McBride and Conte 1996; Allsopp et al. 2011). Ranges for these two species extend from Point Conception, CA to the southern portion of Baja California (National Marine Fisheries Service 2008). As a result of their high market value and demand as an established delicacy, abalone are attractive aquaculture species (Lafarga de la Cruz and Gallardo-Escárate 2011; Moodley et al. 2014).

Although abalone aquaculture is rapidly growing, it has been hindered by several bottlenecks that limit production. Improvements in disease resistance and the other economically important traits will help to reduce production costs and to accelerate growth of the abalone industry, particularly in the United States (Arai and Okumura 2013). The ability to overcome these bottlenecks will be enabled with better genomic resources. When correctly applied, genetic information and techniques such as Marker Assisted Selection can rapidly improve broodstock selection, characterize variation (both beneficial and detrimental), and provide methods to directly improve the value, efficiency, and production in a target species (van der Merwe et al. 2011; Rhode et al. 2012). These tools and techniques may become especially important as aquaculture efforts seek to maintain sustainability in the face of changing ocean conditions. Although there is one related genome assembly published, *Haliotis discus hannai* (Nam et al. 2017) and one currently in preparation *Haliotis laevigata* (Carr 2015), both of these assemblies are highly fragmented and only the *H. discus hannai* genome is publicly available.

Genomic resources are important not only for aquaculture improvement and sustainability but also for enabling conservation efforts (McMahon et al. 2014). White abalone (*H. sorenseni*) was the first invertebrate to be listed as endangered under the US Endangered Species Act in 2001, followed by black abalone (*H. cracherodii*) in 2009. Pinto abalone (*H. kamtschatkana*) was listed as a Species of Concern in the United States in 2004, and as endangered under Canada’s Species at Risk Act since 2009. Ongoing research efforts seek to establish healthy cultures of these species in captivity, with the eventual goal of restoration of natural populations. This effort may be directed and enhanced with genome-enabled tools.

Here, we report a high-quality reference genome assembly and annotation for red abalone, including an examination of genome-wide single nucleotide polymorphism (SNP) variation for five related abalone species native to the Pacific coast of North America: the green, pink, pinto, black, and white abalone.

## Materials and Methods

### Sample Collection and Sequencing

DNA was extracted using the DNeasy Blood and Tissue Kit (Qiagen, Germantown, MD) following the manufacturer’s protocol from the gill tissue of a male and female red abalone specimen, from the epipodial tissues of male and female green, pink, white, and black abalone, and from the epipodial and mantle tissues from a male and female pinto abalone. Dovetail Chicago Illumina HiSeq 3000 and Pacific Biosciences (PacBio) RSII data were generated for only the female red abalone, whereas 150-bp paired-end (insert size ∼500 bp) read and 150-bp mate-pair (insert size ∼15 kb) read Illumina data were generated for both male and female red abalone. Each library corresponds to one individual. Paired-end 150-bp Illumina resequencing data were obtained for male and female specimens for each resequenced species (supplementary Note 8, Supplementary Material online).

### Assembly Strategy

**Fig. 2.**
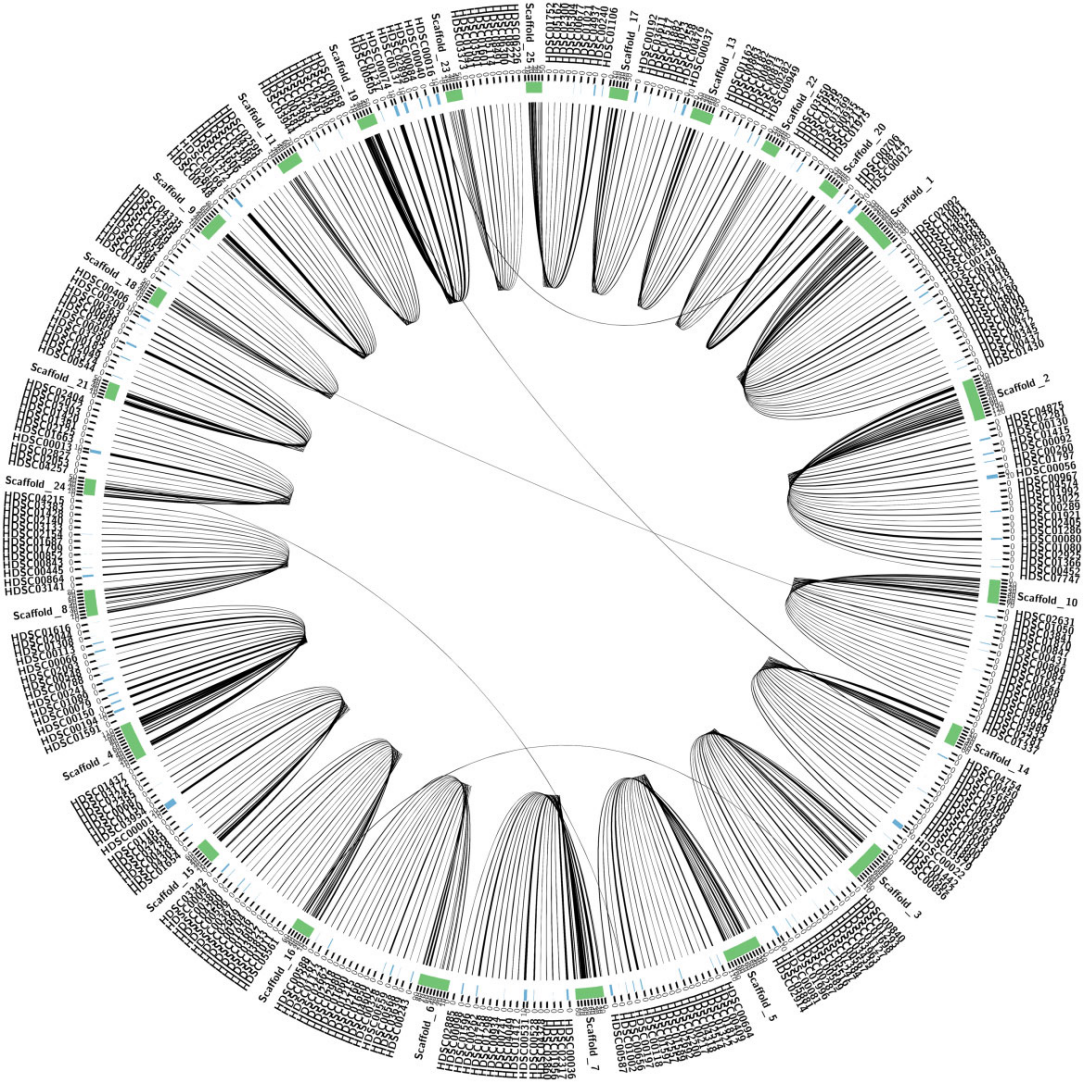
—Synteny between *Haliotis rufescens* (green) and *Haliotis discus hannai* (blue) highlighting the greater continuity in *H. rufescens*.

Raw data were quality checked with FastQC (Andrews 2010) prior to assembly. Trimming for quality and adapters was performed with Quorum (Marçais et al. 2015), which is built into the MaSuRCA assembly pipeline. An initial genome assembly was generated with MaSuRCA version 3.2.2 (Zimin et al. 2013), using paired-end (75× coverage), mate-pair (148× coverage) reads from both male and female samples, and PacBio (29× coverage) reads generated for the female sample. The following parameters were set apart from default: jellyfish hash size (JF_SIZE = 20,000,000,000), paired-end insert size and standard deviation (250, 50), and mate-pair insert size and standard deviation (15,000, 1,000). This initial assembly was scaffolded using long-range distance information obtained from Chicago in vitro proximity ligation libraries (7× expected coverage) with the proprietary HiRise2 program version v2.1.2-ad17ecf8bf57 (Dovetail, Santa Cruz, CA; Putnam et al. 2016). Scaffolds ≤150 bp were removed. Contamination was assessed using BlobTools (v0.9.19, Laetsch and Blaxter 2017) with default parameters, and MegaBLAST version 2.6.0 with an upper *e*-value threshold of 1e^−5^ (Zhang et al. 2000) to the NCBI nr/nt database downloaded on September 17, 2016 (supplementary Note 3, Supplementary Material online). Synteny between *H.rufescens* (green) and *H.discus hannai* (blue) for figure 2 was visualized using Circos (Krzywinski et al. 2009). To obtain syntenic relationships, the following steps were performed: 1) GMAP (Wu and Nacu 2010) was used to map *H. rufescens* genes to the *H. discus hannai* genome downloaded from http://gigadb.org/dataset/100281; last accessed January 23, 2019, 2) Opscan (Drillon et al. 2014) with fastp (Chen et al. 2018) and global alignments as inputs was used to generate ortholog families between the two gene sets, with only primary alignments considered in *H. discus hannai*, and 3) i-ADHoRe 3.0.01 (Proost et al. 2012) was used with the following parameters: prob_cutoff = 0.001, level 2 multiplicons only, cluster_gap = 20, gap_size = 15, q_value = 0.05, and minimum of three anchor points to generate the multiplicons.

### Genome Assembly Quality Assessment

The quality of the genome was assessed by estimating the percent of raw data that aligned to the assembly and by the number of metazoan genes with BUSCO version 3.2.2 using default parameters (Simão et al. 2015). Paired-end and mate-pair raw data alignment was performed using GSNAP version 20170317 (Wu and Nacu 2010) for Illumina data and BLASR version 5.1 (Chaisson and Tesler 2012) for PacBio data. The greater the number of Universal Single Copy Orthologs and the higher the percent of raw data realignment are indicative of a more complete assembly.

### Annotation Strategy

Genome sequences for transcript and protein homology gene predictions were obtained from NCBI for the following species: *Crassostrea gigas* (GCA_000297895.1, Zhang et al. 2012), *Crassostrea virginica* (GCA_002022765.4), *Mytilus galloprovincialis* (GCA_001676915.1, Murgarella et al. 2016), and *Mizuhopecten yessoensis* (GCA_002113885.2, Wang 2017). Genomes were downloaded and transcripts were extracted using the gene models in the gff file (supplementary Note 7, Supplementary Material online). Three lanes worth of 150-bp paired-end (insert size ∼300 bp) Illumina HiSeq 3000 RNA-Seq data for *H.rufescens* were obtained from 12 tissues from a single female (cephalic tentacle, epipodium, epipodal tentacle, ganglion, gonad, heart, kidney, liver, foot, gill, mantel, and postesophagus), two tissues from a single male (gonad and light receptor), and from pools of individuals from each of early-life developmental stages (egg, 1 day, 6 days, 7 days [the 7-day time point included a 24-h acute carbon dioxide exposure ∼1,200 ppm and control CO_2_ exposure], 10 days posthatch, and 1 month posthatch). All samples were extracted in duplicate to create replicate libraries.

RNA-Seq read data have been deposited in the NCBI short read archive (BioProject accession: PRJNA488641). The RNA-Seq data were assembled using Trinity version 2.3.2 (Grabherr 2011) with default parameters, and subsequently used as expressed sequence tag (EST) evidence. EST evidence was also gathered from all available Bivalvia ESTs in NCBI on January 26, 2018. MAKER2 version 2.31.8b (Holt and Yandell 2011) was run on the masked genome using all data described as evidence (supplementary Note 7, Supplementary Material online). In the final annotation, function information was added to the predicted gene models. Curated databases, SwissProt/UniProt (UniProt Consortium 2016, accessed October 5, 2017), were used to identify putative function based on BlastP homology with default parameters and an upper *e*-value cutoff of 1e^−5^ (Camacho et al. 2009). Default parameters for InterProScan version 5.26-65.0 (Jones et al. 2014) were applied to searches against the databases that make up the InterPro Consortium.

### Variance and Relatedness of Five Other *Haliotis* Species

**Fig. 3.**
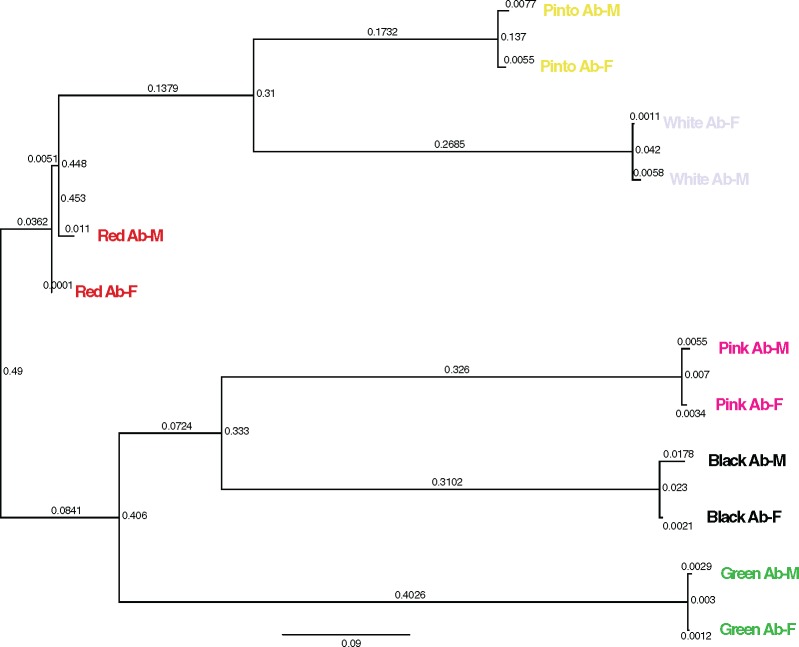
—Unrooted phylogenetic tree indicating relatedness between red abalone (*Haliotis rufescens*), green abalone (*H. fulgens*), black abalone (*H. cracherodii*), white abalone (*H. sorenseni*), pink abalone (*H. corrugata*), and pinto abalone (*H. kamtschatkana*). M and F stand for male and female.

Raw sequences from ten abalone samples (two from each of five species) were quality checked with FastQC (Andrews 2010). Reads were aligned to the *H. rufescens* genome assembly using BWA-MEM version 0.7.17 with default parameters (Li 2013). The BAM files were sorted (samtools sort), cleaned (picard CleanSam), marked for duplicates (picard MarkDuplicates with REMOVE_DUPLICATES=true), read groups were added (picard AddOrReplaceReadGroups), and SNP/InDel realignment (GATK RealignerTargetCreator) was performed with Picard Tools 2.4.1 (https://broadinstitute.github.io/picard/; last accessed January 23, 2019) and GATK prior to calling SNPs and InDels with the HaplotypeCaller function in GATK version 3.5 (McKenna 2010). Default parameters were used unless otherwise specified. SNPs were filtered with VCFtools version 0.1.14 (Danecek 2011) using the following parameters (–max-non-ref-af 0.8 –non-ref-af 0.2 –hwe 0.001). The phylogenetic tree in figure 3 was generated with the filtered SNPs using SNPhylo version 2016-02-04 (Lee et al. 2014) using the maximum likelihood method. SNPhylo applies a filter to reduce SNP redundancy by linkage disequilibrium (-b -B 100). Visualization of figure 3 was performed in FigTree v1.4.3 (http://tree.bio.ed.ac.uk/software/figtree/; last accessed January 23, 2019).

### Data Availability

The following files: GenomeScope histo files, MAKER2 control files, and Newick tree file can be found at the github repository for this paper (https://github.com/ISUgenomics/RedAbaloneGenomePaper_GBE_2018; last accessed January 23, 2019). The genome assembly, annotation and VCF files can be found at https://abalone.dbgenome.org, last accessed January 23, 2019. The NCBI genome ID is 16745.

## Results and Discussion

### Genome Assembly

The *H. rufescens* genome was assembled with MaSuRCA resulting in an initial assembly of 12,918 scaffolds with a N50 of 588 kb (supplementary Note 1, Supplementary Material online). Using long distance information obtained from the Chicago library data, HiRise2 was able to improve the contiguity of the assembly and reduce the number of scaffolds to 8,371 for the 1.498 Gb, with a scaffold N50 of 1.895 Mb (supplementary Note 1, Supplementary Material online). A difference of ∼300 Mb from the estimated genome size of 1.8 Gb (Gallardo-Escarate and del Rio-Portilla 2007) may be attributed to a lack of large repeat-spanning reads (supplementary Note 2, Supplementary Material online) resulting in the collapse of some of the repeat regions. Although assembly contiguity has not yet been resolved into chromosomes in *H. rufescens*, the current assembly represents a 10-fold improvement in contiguity compared with other existing abalone genomes for *H.discus hannai* (Nam et al. 2017) and *H.laevigata* (Carr 2015) with 80,032 scaffolds at a N50 of 200 kb and 105,431 scaffolds at a N50 of 81 kb, respectively.

### Genome Assembly Quality Assessment

To assess the quality and completeness of the genome assembly, the raw sequence data were aligned to the draft assembly. A high percentage of raw Illumina reads;:96%, 74%, 91%, and 86% of the paired-end, mate-pair, PacBio, and Chicago data aligned to the assembly, respectively. More information about the quality of the raw data and alignment can be found in supplementary Note 9, Supplementary Material online, and in the github repository for this paper (https://isugenomics.github.io/RedAbaloneGenomePaper_GBE_2018/; last accessed January 23, 2019). A complete spirochaete genome was also identified in the data with BlobTools and removed (supplementary Note 3, Supplementary Material online). To evaluate genic regions of the genome, we estimated the number of BUSCO genes included in our assembly (Benchmarking Universal Single Copy Orthologs). Of the 978 possible metazoan BUSCO genes, 930 (95.1%) were complete, 10 (1%) were fragmented, and 38 (3.9%) were missing (supplementary Note 4, Supplementary Material online). In addition, 679 scaffolds in the *H. rufescens* genome had genes that were in synteny with 320 Mb in 1, 816 scaffolds in the *H. discus hannai* genome, covering a total of 384 Mb of 1,151 Mb in the *H. rufescens* genome. From the Circos plot displaying the synteny, we see that the genome assembly of *H. rufescens* is significantly less fragmented than the genome assembly of *H. discus hannai* as determined by a large number of *H. discus hannai* scaffolds that are syntenic to individual *H. rufescens* scaffolds (fig. 2). Altogether, the high proportion of raw read mapping, a high BUSCO completeness, and a reasonably high degree of synteny suggests the *H. rufescens*’ genome assembly is of high quality.

### Genome Annotation

The annotation resulted in 57,785 gene models where gene length averaged 8,255 bp. Most of these genes (57,771) revealed high levels of evidence for the gene model, represented by an Annotation Edit Distance (AED) score of <1; a score of 1 indicates very little evidence, whereas a score of 0 indicates substantial evidence for a particular gene model. In fact, 87% of annotated genes had AED scores <0.6 (supplementary Note 5, Supplementary Material online). A total of 43,795 genes had a functional annotation with BLAST to the SwissProt/UniProt database, whereas 28,335 had domain hits to the InterProScan database. The number of predicted gene models is similar to the number of genes identified by the International Abalone Genome Consortium for *H.laevigata* (55,000 gene models, Carr 2015), however, it is almost twice as many as were annotated in *H.discus hannai* (29,449 gene models, Nam et al. 2017). Differences in gene model prediction strategies and in potential gene model fragmentation may explain some of these discrepancies.

### Variants, Heterozygosity, and Repetitiveness

GATK was used to call 96,084,900 SNPs among six abalone species, with each species represented by two individuals. A filtered subset containing 1,784,991 SNPs were used to construct a phylogenetic tree (fig. 3), confirming, as expected, that male and female individuals from each species cluster together. As sequence data for the five abalone species were aligned to the red abalone genome, both red abalone specimens appear as short branches roughly in the middle of this unrooted tree. Phylogenetic relationships largely follow patterns detected by previous studies (Gruenthal and Burton 2006; Crosson and Friedman 2018). Relatively shallow divergence between red, white, and pinto abalone is observed with the least divergence between pinto and white abalone. Pink, black, and green abalone exhibit deeper levels of divergence (fig. 3). Genomic phylogenetic data may be useful for efforts, such as white abalone restoration, as closely related species may serve as experimental models for examining disease (Crosson and Friedman 2018) and environmental parameters relevant to culturing and outplanting efforts for this and other endangered species.

Estimates of heterozygosity and repetitiveness were measured from the raw paired-end Illumina data using GenomeScope (supplementary Note 6, Supplementary Material online). Levels of repetitiveness ranged from 33% to 39.3%, which corresponds to the level estimated from RepeatMasker (33%, supplementary Note 2, Supplementary Material online). Estimated heterozygosity for each of the six species ranged from 0.43% to 1.04%, with black and pinto abalone exhibiting the highest levels of heterozygosity with 1.04 and 1.02, respectively. White abalone were the least heterozygous with a value of 0.43, presumably due to the samples originating from F2 generation full siblings in culture. Green, pink, and red abalone had values of 0.68, 0.76, and 0.95, respectively. Research is ongoing to evaluate genetic diversity in wild and cultured white abalone to help guide restoration culture efforts and maximize remaining levels of genetic diversity in this species. These values of heterozygosity are lower than those found in other mollusks *Bathymodiolus platifrons* (1.24), *Modiolus philippinarum* (2.02), and *Chlamys farreri* (1.4) (Jiao et al. 2014; Sun et al. 2017)*.* Heterozygosity in the cultured species *Argopecten irradians* had values more in line with what was determined in *Haliotis* with a heterozygosity value of 0.9 (Du et al. 2017). This lower level of heterozygosity in wild abalone is likely due to the significant loss of wild populations of the Californian populations due to withering syndrome (Crosson and Friedman 2018).

## Conclusion

Here, we present the annotated draft genome for red abalone, *H.rufescens*. This draft genome represents the most contiguous abalone genome assembly available to date. It will also serve as a valuable resource for future genomic research to improve commercial red abalone culture and to inform conservation strategies for the endangered white and black abalone.

## Supplementary Material

Supplementary data are available at *Genome Biology and Evolution* online.

## Supplementary Material

Supplementary Data

## Footnotes

**Data deposition:** Raw data along with the genome assembly are submitted at NCBI under the BioProject ID PRJNA434455 and PRJNA488641.
