# Supplementary material for: An Annotated Genome for Haliotis rufescens (Red Abalone) and Resequenced Green, Pink, Pinto, Black, and White Abalone Species
Source: Genome Biol Evol. 2019 Jan 17;11(2):431–8. doi: 10.1093/gbe/evz006 (PMC6373831; doi:10.1093/gbe/evz006)
Supplement: Supplementary Data [file evz006_supp.docx]

**Supplemental Note 1. Assembly statistics**

Assembly statistics generated from the new_assemblathon.pl script after MaSuRCA 3.2.2, MaSuRCA 3.2.2 plus HiRise2, MaSuRCA 3.2.2 plus HiRise2 and removing scaffolds >150bp, MaSuRCA 3.2.2, HiRise2, removing scaffolds >200bp and Scaffold_85, a spirochaete genome.

| Assembly | MaSuRCA | MaSuRCA + Chicago | MaSuRCa + Chicago + 150bp filter | MaSuRCa + Chicago + 150bp filter + NCBI filter |
| --- | --- | --- | --- | --- |
| Assumed genome size (Mb) | 1779.96 | 1779.96 | 1779.96 | 1779.96 |
| Number of Scaffolds | 12,918 | 8,496 | 8,405 | 8,371 |
| Assembled genome size | 1,495,992,103 | 1,501,971,245 | 1,501,960,693 | 1,498,703,277 |
| Assembled genome size as percentage of assumed genome size | 84.0% | 84.4% | 84.4% | 84.2% |
| Longest scaffold (bp) | 5,901,594 | 13,188,215 | 13,188,215 | 13,188,215 |
| Shortest scaffold (bp) | 64 | 64 | 154 | 203 |
| Number of scaffolds >1kb | 12,752 | 8,330 | 8,330 | 8,329 |
| Number of scaffolds >10kb | 6,155 | 3,034 | 3,034 | 3,033 |
| Number of scaffolds >100kb | 3,080 | 1,466 | 1,466 | 1,465 |
| Number of scaffolds >1Mb | 262 | 424 | 424 | 423 |
| Number of scaffolds >10Mb | 0 | 5 | 5 | 5 |
| Mean scaffold size (bp) | 115,807 | 176,786 | 178,698 | 179,035 |
| Median scaffold size | 8,502 | 4,536 | 4,629 | 4,661 |
| N50 scaffold length | 588,068 | 1,897,768 | 1,897,768 | 1,895,871 |
| L50 scaffold count | 709 | 214 | 214 | 214 |
| Scaffold % A | 29.38 | 29.26 | 29.26 | 29.27 |
| Scaffold % C | 20.18 | 20.10 | 20.10 | 20.09 |
| Scaffold % G | 20.20 | 20.13 | 20.13 | 20.12 |
| Scaffold % T | 29.40 | 29.29 | 29.29 | 29.30 |
| Scaffold % N | 0.83 | 1.22 | 1.22 | 1.22 |
| Scaffold %non-ACGTN bp | 0.0 | 0.0 | 0.0 | 0.0 |
| % of assembly in scaffolds | 56.0 | 93.6 | 93.6 | 93.8 |
| % of assembly in unscaffolded contigs | 44.0 | 6.4 | 6.4 | 6.2 |
| Average number of contigs per scaffold | 1.3 | 2.1 | 2.2 | 2.2 |
| Average length of breaks (>25Ns) between contigs in scaffolds | 3,265 | 1,876 | 1,876 | 1,876 |

**Supplemental Note 2. RepeatModeler and RepeatMasker results**

|  | **Number of elements** | **Base pairs** | **Percentage of genome** |
| --- | --- | --- | --- |
| Total masked | NA | 496,570,997 | 33.06% |
| SINEs | 720,033 | 103,219,025 | 6.87% |
| ALU | 0 | 0 | 0.00% |
| MIR | 4,162 | 536,097 | 0.04% |
| LINEs | 533,232 | 132,993,467 | 8.85% |
| LINE1 | 4,528 | 979,644 | 0.07% |
| LINE2 | 0 | 0 | 0.00% |
| L3/CR1 | 0 | 0 | 0.00% |
| LTR elements | 23,980 | 10,504,053 | 0.70% |
| ERVL | 0 | 0 | 0.00% |
| ERVL-MaLRs | 0 | 0 | 0.00% |
| ERV_class I | 0 | 0 | 0.00% |
| ERV_class II | 0 | 0 | 0.00% |
| DNA elements | 180,300 | 72,142,926 | 4.80% |
| hAT-Charlie | 0 | 0 | 0.00% |
| TcMar-Tigger | 5,519 | 629,038 | 0.04% |
| Unclassified | 607,953 | 147,591,890 | 9.83% |
| Total interspersed repeats | NA | 466,451,361 | 31.06% |
| Small RNA | 203,302 | 24,731,383 | 1.65% |
| Satellites | 11,527 | 2,126,513 | 0.14% |
| Simple repeats | 325,198 | 26,864,292 | 1.79% |
| Low complexity | 19,194 | 1,487,993 | 0.10% |

**Supplemental Note 3. Contamination check of assembly MaSuRCA + Chicago with Blobtools**

Contamination in the genome assembly was checked prior to submission to NCBI. Scaffold_85 was removed as it is a fully assembled spirochaete. All other potential contamination was determined to be false positives with either the 2nd best BLAST hit to the *Haliotis* genus or represented a repeat sequence with a low level of homology to the potential contaminant.


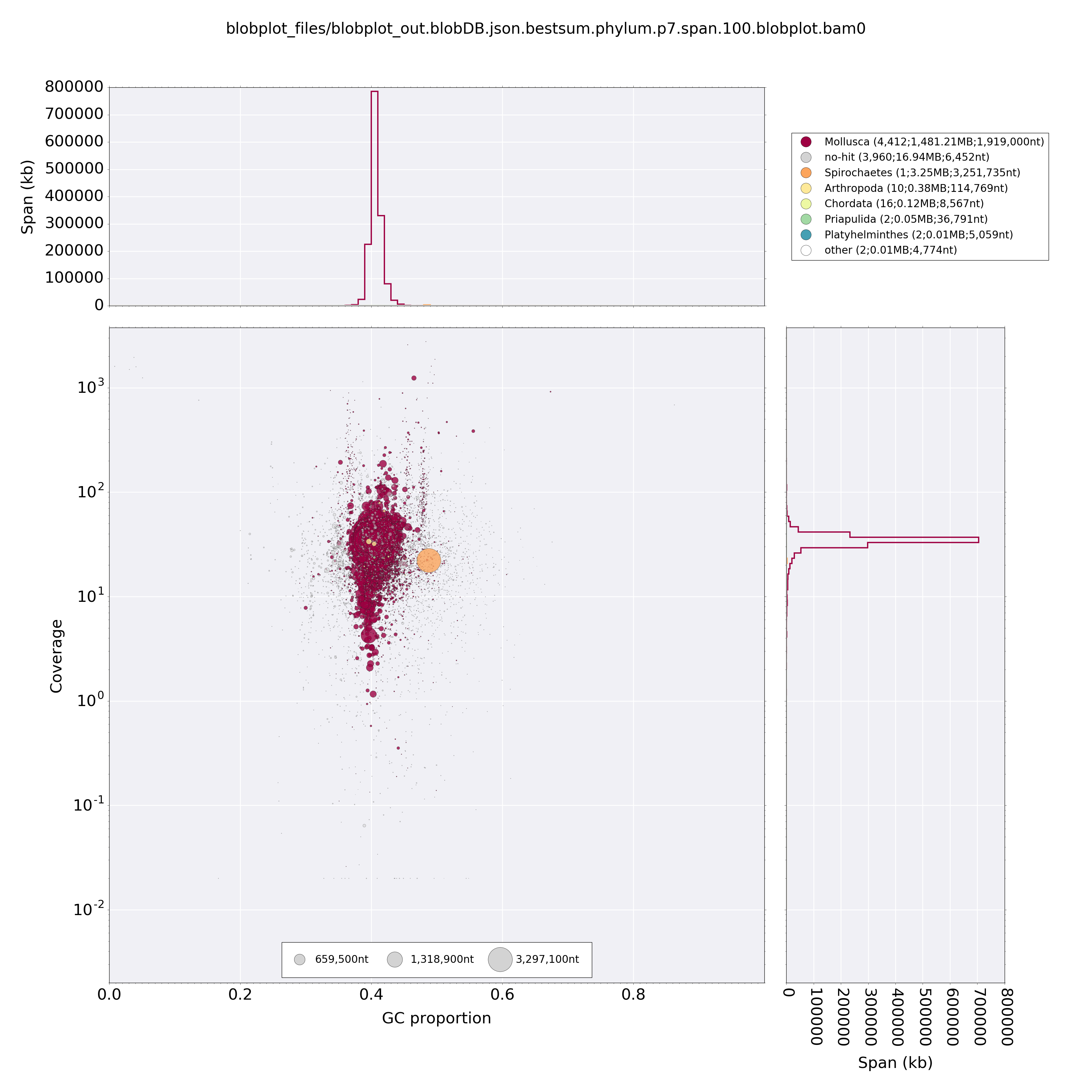


**Supplemental Note 4. BUSCO**

BUSCO v3 was run in genome mode. Only a small number of genes were missing, suggesting the abalone genome assembly is nearly complete.

|  | Total BUSCO genes | Percent of total BUSCOs searched |
| --- | --- | --- |
| Complete BUSCOs (C) | 930 | 95.1% |
| Complete and single-copy BUSCOs (S) | 867 | 88.7% |
| Complete and duplicated BUSCOs (D) | 63 | 6.4% |
| Fragmented BUSCOs (F) | 10 | 1.0% |
| Missing BUSCOs (M) | 38 | 3.9% |
| Total BUSCO groups searched | 978 | 100% |

**Supplemental Note 5. Cumulative sum of AED scores**

A representation of gene quality is shown by plotting AED score vs gene frequency. As AED increases, a gene’s transcriptional support declines. Thus, most of the genes in the *H. rufescens* genome have high transcriptional support.


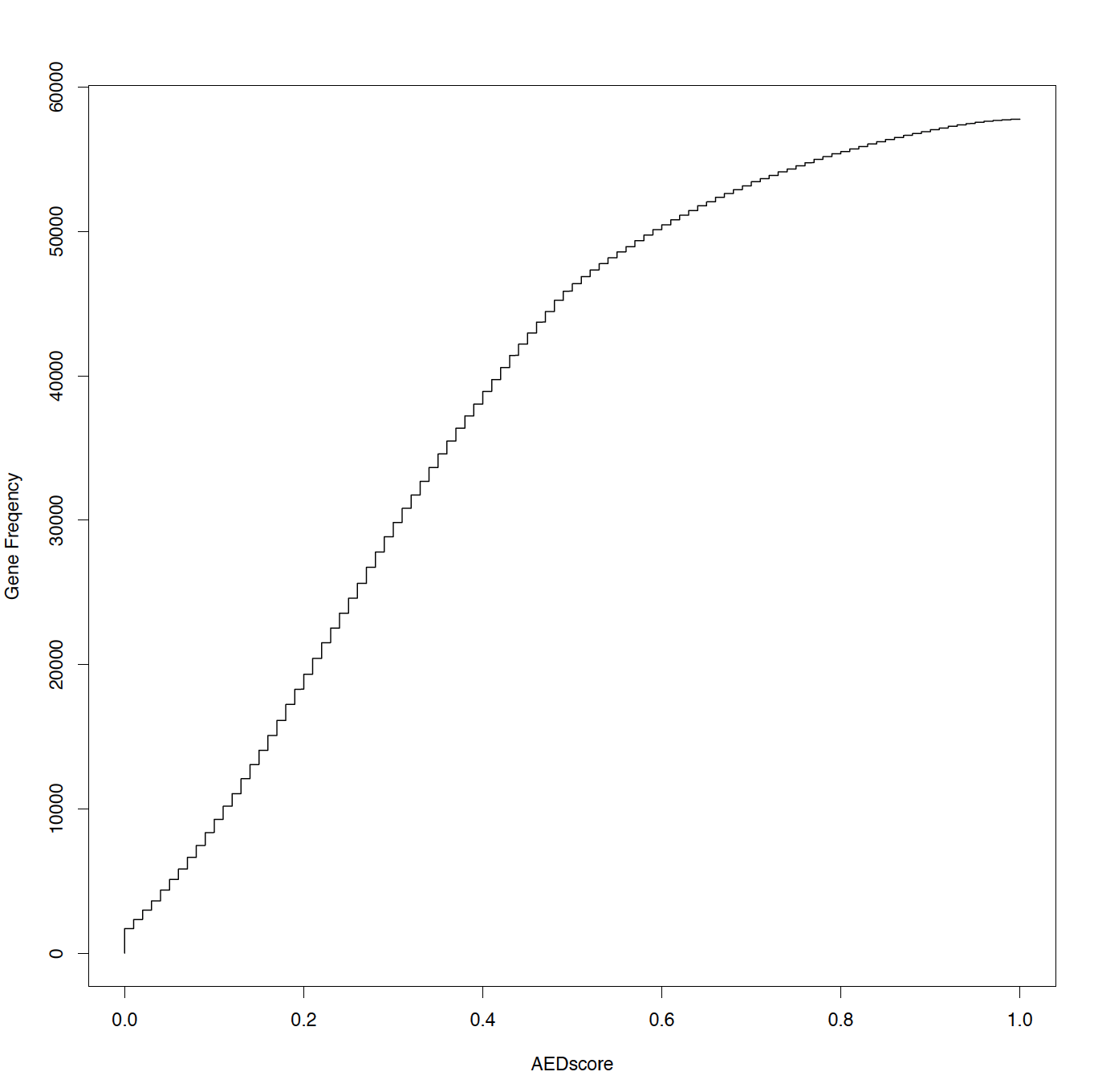


**Supplemental Note 6. GenomeScope and heterozygosity determination**

The following script was run on the raw Illumina data for each species. The .histo files were then uploaded to the GenomeScope server (<http://qb.cshl.edu/genomescope/>). GenomeScope version 1 for a kmer value of 21.

runGenomeScope.sh

#!/bin/bash

module load jellyfish/2.2.5

kmer=$1

a1=XXX-Ab-F_S21_L005_R1_001.fastq.gz

a2=XXX-Ab-F_S21_L005_R2_001.fastq.gz

b1=XXX-Ab-M_S22_L005_R1_001.fastq.gz

b2=XXX-Ab-M_S22_L005_R2_001.fastq.gz

jellyfish count -C -m $kmer -s 1000000000 -t 10 <(zcat ${a1}) <(zcat ${a2}) <(zcat ${b1}) <(zcat ${b2}) -o reads_K${kmer}.jf

jellyfish histo -t 16 reads_K${kmer}.jf > reads_K${kmer}.histo

GenomeScope results are displayed below for each species.


|  | **property** | **Heterozygosity** | | **Genome Haploid Length** | | **Genome Repeat Length** | | **Genome Unique Length** | **Model Fit** | **Read Error Rate** |
| --- | --- | --- | --- | --- | --- | --- | --- | --- | --- | --- |
| Black | min | 1.04% | | 1,043,004,213 bp | | 344,565,334 bp | | 698,438,879 bp | 94.56% | 1.38% |
|  | max | 1.05% | | 1,044,283,049 bp | | 344,987,808 bp | | 699,295,240 bp | 99.16% | 1.38% |
| Green | min | 0.68% | | 1,058,596,325 bp | | 340,592,180 bp | | 718,004,145 bp | 94.70% | 1.09% |
|  | max | 0.69% | | 1,060,046,435 bp | | 341,058,738 bp | | 718,987,698 bp | 99.11% | 1.09% |
| Pink | min | 0.76% | | 1,051,384,798 bp | | 336,470,057 bp | | 714,914,741 bp | 94.47% | 1.40% |
|  | max | 0.80% | | 1,053,496,551 bp | | 337,145,872 bp | | 716,350,679 bp | 99.33% | 1.40% |
| Pinto | min | 1.02% | | 1,105,527,543 bp | | 382,795,357 bp | | 722,732,185 bp | 94.17% | 0.80% |
|  | max | 1.03% | | 1,106,852,445 bp | | 383,254,112 bp | | 723,598,332 bp | 99.17% | 0.80% |
| White | min | 0.43% | | 1,213,288,263 bp | | 476,230,427 bp | | 737,057,836 bp | 93.74% | 0.87% |
|  | max | 0.44% | | 1,214,431,776 bp | | 476,679,270 bp | | 737,752,506 bp | 99.28% | 0.87% |
| Red | min | 0.95% | | 1,158,394,126 bp | | 438,760,994 bp | | 719,633,132 bp | 93.56% | 0.86% |
|  | max | 0.96% | | 1,160,439,877 bp | | 439,535,856 bp | | 720,904,021 bp | 98.78% | 0.86% |

**Supplemental Note 7.**

Annotation with MAKER2

Gene annotation was accomplished using MAKER2 in four successive rounds. Round one used a RepeatModeler/Repeatmasker masked genome using the unchanged version of the repeat database from Repbase included with MAKER2 and MAKER2’s internal homology-based gene prediction algorithm, all available Bivalvia EST’s from NCBI were used along with the proteins and CDS from the genomes of *Crassostrea gigas*, *Crassostrea virginica*, *Mytilus galloprovincialis*, and *Mizuhopecten yessoensis*. Protein and CDS sequences were extracted from the genome assemblies using their corresponding GFF files and gff2fasta.pl (<https://github.com/ISUgenomics/common_scripts/blob/master/gff2fasta.pl>). Round two of MAKER2 used the same internal algorithm with ~2 million *Haliotis rufescens* transcripts from a de-novo Trinity transcriptome. The SNAP gene predictor was utilized the MAKER2-derived gff in a third round of MAKER2. A fourth round of MAKER2 utilized GeneMark ES predictions and Augustus training derived from BUSCO. In MAKER2 rounds 2-4, the previously generated gff file is supplied as input using the MAKER2_gff option in the control file. See github repository for more details (https://isugenomics.github.io/RedAbaloneGenomePaper_GBE_2018/)

**Supplemental Note 8**

Specimen Collection

Sexing of abalone is difficult when gonads are not fully mature, when possible visually determined sex was confirmed by induced gamete expression. Male and female (sexed by gamete expression), captive bred, red abalone (*H. rufescens*) specimens were provided by a commercial abalone producer, The Cultured Abalone (Goleta, CA). Gill tissue was extracted from these individuals and immediately placed in liquid nitrogen until DNA extraction. Epipodiums from male and female (sexed by gamete expression) green (*H. fulgens*), pink (*H. corrugata*), and black (*H. cracherodii*, sexed visually) abalone were collected, non-lethally, from specimens held in culture at the NOAA Fisheries, Southwest Fisheries Science Center (La Jolla, CA. see table below for relevant permit information). The sampled tissues were immediately placed in 100% ethanol until DNA extraction. Epipodial samples from sexed (sexed by gamete expression) white abalone (*H. sorenseni*) were acquired from the Aquarium of the Pacific (Long Beach, CA) and Bodega Marine Lab (Bodega, CA. see relevant permit information in table below). Epipodial and mantle tissue were collected from sexed (sexed visually) pinto abalone (*H. kamtschatkana)* provided by the Puget Sound Restoration Fund (Bainbridge Island, WA), these samples were frozen at -80 °C until DNA extraction.

Genomic DNA Preparation

For 150 bp paired-end and 150 bp mate-pair (insert size ~ 15kb) Illumina sequencing applications (red, pink, green, black, white, and pinto abalone), genomic DNA was extracted using the DNeasy Blood and Tissue Kit (Qiagen, Germantown, MD) following the manufacturer’s protocol. The purity of genomic DNA was assessed with a NanoDrop ND-1000 spectrophotometer and DNA quantification was performed using a Qubit Fluorometer (ThermoFisher Scientific, Waltham, MA). Samples prepared for the Illumina genomic and re-sequencing applications were sent to the DNA Sequencing Facility at Iowa State University (Ames, Iowa) for library preparation and sequencing.

Red abalone gill tissue was sent to Dovetail Genomics (Santa Cruz, CA) for high molecular weight (HMW) genomic DNA extraction using a Qiagen Genomic DNA extraction kit, following the manufacturer’s protocol. Mean DNA fragment size was 100kb, and subsequently applied to produce long-range sequencing libraries at Dovetail Genomics and PacBio libraries at Iowa State University. All data was sequenced on either the Illumina HiSeq 3000 or the PacBio RSII in the DNA facility at Iowa State University.

Specimen information for the six abalone species sequenced, including, individual tag IDs, sex of the specimen, the collection source, associated permit numbers (if applicable), notes about the specimen, tissue type for DNA extraction, 150bp paired-end reads generated, and genomic fold coverage.

| **Species** | **Individual ID** | **Sex** | **Source and permit Number (if applicable)** | **Specimen notes** | **Tissue for DNA extraction** | **Reads Generated (150bp)** | **Coverage** |
| --- | --- | --- | --- | --- | --- | --- | --- |
| **Black abalone** |  |  |  |  |  |  |  |
| *H. cracherodii* | Halcra 3kk57 | F (visual) | Tissue collected from individual held at the Southwest Fisheries Science Center (La Jolla, CA); Black Abalone ESA Permit #19571 | Wild-caught broodstock individual | Epipodium | 350,089,235 | 35.0x |
| *H. cracherodii* | Halcra 2notag | M (visual) | Tissue collected from individual held at the Southwest Fisheries Science Center (La Jolla, CA); Black Abalone ESA Permit #19571 | Wild-caught broodstock individual | Epipodium | 256,234,656 | 25.6x |
| **Green abalone** |  |  |  |  |  |  |  |
| *H. fulgens* | 807-60 | M | Tissue collected from individual held at the Southwest Fisheries Science Center (La Jolla, CA); CDFW Permit SCP #12372 | Wild-caught broodstock individual | Epipodium | 349,310,180 | 34.9x |
| *H. fulgens* | 807-53 | F | Tissue collected from individual held at the Southwest Fisheries Science Center (La Jolla, CA); CDFW Permit SCP #12372 | Wild-caught broodstock individual | Epipodium | 267,065,407 | 26.7x |
| **Pink abalone** |  |  |  |  |  |  |  |
| *H. corrugata* | PinkAb 104 | F | Tissue collected from individual held at the Southwest Fisheries Science Center (La Jolla, CA); CDFW Permit SCP #12372 | Wild-caught broodstock individual | Epipodium | 288,477,903 | 28.8x |
| *H. corrugata* | PinkAb 080 | M | Tissue collected from individual held at the Southwest Fisheries Science Center (La Jolla, CA); CDFW Permit SCP #12372 | Wild-caught broodstock individual | Epipodium | 347,556,275 | 34.7x |
| **Pinto Abalone** |  |  |  |  |  |  |  |
| *H. kamtschatkana* | PinAb 32 M | M (visual) | Tissue collected from individuals provided by the Puget Sound Restoration Fund (Bainbridge Island, WA) | Captive bred F1, and full-sib of the female specimen | Epipodium and mantle | 195,269,246 | 19.5x |
| *H. kamtschatkana* | PinAb 55 F | F (visual) | Tissue collected from individuals provided by the Puget Sound Restoration Fund (Bainbridge Island, WA) | Captive bred F1, and full-sib of the male specimen | Epipodium and mantle | 287,010,556 | 28.7x |
| **Red abalone** |  |  |  |  |  |  |  |
| *H. rufescens* | RedAb 103-F | F | Tissue collected from an individual provided by the Cultured Abalone (Goleta, CA) | Captive bred F1, relation to other specimen unknown | Gill | 236,367,563 | 23.6x |
| *H. rufescens* | RedAb 105-M | M | Tissue collected from an individual provided by the Cultured Abalone (Goleta, CA) | Captive bred F1, relation to other specimen unknown | Gill | 396,627,872 | 39.6x |
| **White abalone** |  |  |  |  |  |  |  |
| *H. sorenseni* | AOP033 | M | Tissue collected from individual held at the Aquarium of the Pacific (Long Beach, CA); White Abalone ESA Permit #14344-2R | Captive bred F1, and full-sib of the female specimen | Epipodium | 298,977,986 | 29.9x |
| *H. sorenseni* | BML328 green | F | Tissue collected from individual held at the Bodega Marine Lab (Bodega, CA); White Abalone ESA Permit #14344-2R | Captive bred F1, and full-sib of the male specimen | Epipodium | 312,392,699 | 31.2x |

**Supplemental Note 9.**

Picard QualityScoreDistribution with default parameters was used to generate this table for each of the Abalone species. It shows that that the majority of the alignments have high quality base scores.

| **QUALITY/ COUNT_OF_Q** | **8** | **12** | **22** | **27** | **32** | **37** | **41** |
| --- | --- | --- | --- | --- | --- | --- | --- |
| **Black-Ab-F** | 8.90E+08 | 6.62E+09 | 2.77E+09 | 2.16E+09 | 3.79E+09 | 7.25E+09 | 2.90E+10 |
| **Black-Ab-M** | 7.14E+08 | 5.43E+09 | 2.18E+09 | 1.63E+09 | 2.80E+09 | 5.28E+09 | 2.04E+10 |
| **Green-Ab-F** | 1.06E+09 | 7.56E+09 | 2.83E+09 | 1.99E+09 | 3.53E+09 | 6.89E+09 | 2.85E+10 |
| **Green-Ab-M** | 4.52E+08 | 3.51E+09 | 1.75E+09 | 1.54E+09 | 2.87E+09 | 5.62E+09 | 2.43E+10 |
| **Pinto-Ab-M** | 2.07E+08 | 2.10E+09 | 1.08E+09 | 1.05E+09 | 2.05E+09 | 4.00E+09 | 1.83E+10 |
| **Pinto-Ab-F** | 3.35E+08 | 3.31E+09 | 1.68E+09 | 1.59E+09 | 3.08E+09 | 5.95E+09 | 2.64E+10 |
| **Pink-Ab-F** | 8.88E+08 | 6.21E+09 | 2.35E+09 | 1.65E+09 | 2.94E+09 | 5.62E+09 | 2.36E+10 |
| **Pink-Ab-M** | 8.85E+08 | 6.63E+09 | 2.78E+09 | 2.08E+09 | 3.70E+09 | 7.01E+09 | 2.90E+10 |
| **Red-Ab-F** | 3.30E+08 | 2.83E+09 | 1.49E+09 | 1.30E+09 | 2.45E+09 | 4.79E+09 | 2.23E+10 |
| **Red-Ab-M** | 6.16E+08 | 5.18E+09 | 2.46E+09 | 2.03E+09 | 3.87E+09 | 7.64E+09 | 3.77E+10 |
| **White-Ab-F** | 4.47E+08 | 5.04E+09 | 2.78E+09 | 2.74E+09 | 4.83E+09 | 8.46E+09 | 2.14E+10 |
| **White-Ab-M** | 4.07E+08 | 3.63E+09 | 2.03E+09 | 2.00E+09 | 3.88E+09 | 7.31E+09 | 2.47E+10 |
